# Diagnostic and Prognostic Role of Circulating microRNAs in Patients with Coronary Artery Disease—Impact on Left Ventricle and Arterial Function

**DOI:** 10.3390/cimb46080500

**Published:** 2024-08-03

**Authors:** Loredana Iacobescu, Andrea Olivia Ciobanu, Razvan Macarie, Mihaela Vadana, Letitia Ciortan, Monica Madalina Tucureanu, Elena Butoi, Maya Simionescu, Dragos Vinereanu

**Affiliations:** 1 Department of Cardiology, University of Medicine and Pharmacy Carol Davila, Dionisie Lupu Street, 126900 Bucharest, Romania; loredana.gheorghiu@drd.umfcd.ro (L.I.); andreaciobanu7@yahoo.com (A.O.C.); 2 University and Emergency Hospital, Splaiul Independentei 169, 050098 Bucharest, Romania; 3 Institute of Cellular Biology and Pathology Nicolae Simionescu, 050568 Bucharest, Romania; razvan.macarie@icbp.ro (R.M.); mihaela.vadana@icbp.ro (M.V.); letitia.ciortan@icbp.ro (L.C.); monica.pirvulescu@icbp.ro (M.M.T.); elena.dragomir@icbp.ro (E.B.); maya.simionescu@icbp.ro (M.S.)

**Keywords:** microRNA, myocardial infarction, coronary artery disease, left ventricular function, arterial stiffness

## Abstract

Recent studies reported that circulating microRNAs (miRNAs) can target different metalloproteases (MMPs) involved in matrix remodeling and plaque vulnerability. Consequently, they might have a role in the diagnosis and prognosis of coronary artery disease. To quantify circulating miRNAs (miRNA126, miRNA146, and miRNA21) suggested to have possible cardiovascular implications, as well as levels of MMP-1 and MMP-9, and to determine their association with left ventricular (LV) function and with arterial function, in patients with either ST-segment elevation acute myocardial infarction (STEMI) or stable ischemic heart disease (SIHD). A total of 90 patients with coronary artery disease (61% men, 58 ± 12 years), including 60 patients with STEMI and 30 patients with SIHD, were assessed within 24 h of admission, by measuring serum microRNAs, and serum MMP-1 and MMP-9. LV function was assessed by measuring ejection fraction (EF) by 2D and 3D echocardiography, and global longitudinal strain (GLS) by speckle tracking. Arterial function was assessed by echo tracking, CAVI, and peripheral Doppler. Circulating levels of miRNA146, miRNA21, and MMP1 were significantly increased in patients with STEMI vs. SIHD (*p* = 0.0001, *p* = 0.0001, *p* = 0.04, respectively). MiRNA126 negatively correlated with LVEF (r = −0.33, *p* = 0.01) and LV deformation parameters (r = −0.31, *p* = 0.03) in patients with STEMI and negatively correlated with ABI parameters (r = −0.39, *p* = 0.03, r = −0.40, *p* = 0.03, respectively) in patients with SIHD. MiRNA146 did not have any significant correlations, while higher values of miRNA21 were associated with lower values of GLS in STEMI patients and with higher values of GLS in SIHD patients. Both MMP1 and MMP9 correlated negatively with LVEF (r = −0.27, *p* = 0.04, r = −0.40, *p* = 0.001, respectively) and GLS in patients with STEMI, and positively with arterial stiffness in patients with SIHD (r = 0.40 and r = 0.32, respectively; both *p* < 0.05). MiRNA126, miRNA21, and both MMP1 and MMP9 are associated with LV and arterial function parameters in patients with acute coronary syndrome. Meanwhile, they inversely correlate with arterial function in patients with chronic atherosclerotic disease. However, further studies are needed to establish whether these novel biomarkers have diagnosis and prognosis significance.

## 1. Introduction

Atherosclerosis has been widely studied in the past years, showing that there is permanent damage of the vascular endothelium, with endothelial cell activation, inflammation, and apoptosis [1]. Progress in molecular medicine has revealed an increased plasma level of circulating micro vesicles in atherosclerosis, consequently affecting the endothelial function. These micro vesicles contain bioactive molecules, such us proteins, mRNAs, and microRNAs [1,2].

MicroRNAs (miRNAs) are a class of short noncoding RNAs, which repress post-translational gene expression; the first report that revealed the implication of miRNAs in cardiovascular diseases was published in 2006 [3,4]. A single miRNA is capable of downregulating the expression of hundreds of target genes [5,6]. Studies have shown that miRNAs are involved in proliferation, cell differentiation, apoptosis, and fibrosis [7]. Thus, it has been shown that miRNA126 and miRNA21 have potential implications in coronary artery disease (CAD), while miRNA146 might be involved in the process of atherosclerosis [4,7]. Meanwhile, matrix metalloproteinases (MMPs), released from macrophages, might also be involved in the process of atherosclerosis. MMPs are a family of endopeptidases, calcium-dependent and zinc-containing, able to degrade components of the extracellular matrix, which can cause plaque rupture and instability [8,9,10]. The development of heart failure after myocardial infarction appears as a consequence of left ventricle remodeling, a complex process of molecular, cellular, and interstitial changes. Despite the recent progress in invasive and pharmacological treatment, left ventricle remodeling appears frequently after myocardial infarction, and the most frequent tools to quantify it are left ventricle ejection fraction and left ventricle deformation parameters. Therefore, early detection and prediction of left ventricle remodeling is necessary in order to identify the patients at high risk of heart failure development [10,11]. The diagnostic and prognostic roles of miRNAs and MMPs in CAD need further studies.

Thus, the aim of our study was to quantify circulating miRNAs (miRNA126, miRNA146, and miRNA21), as well as the levels of MMP-1 and MMP-9, and to determine their association with left ventricular (LV) function and with arterial function, in patients with either ST-segment elevation acute myocardial infarction (STEMI) and stable ischemic heart disease (SIHD).

## 2. Materials and Methods

### 2.1. Study Population

We included, prospectively, 90 patients with CAD, including 60 patients with STEMI and 30 patients with SIHD. Inclusion criteria for patients with STEMI were based on the 2017 ESC/AHA/ACC guidelines, while for patients with SIHD, these were based on the 2019 ESC guidelines [11,12]. Other inclusion criteria were age > 18 years and sinus rhythm. Patients with severe heart failure or other significant medical conditions were excluded. Patients were assessed within 24 h of admission by serum high-sensitive troponin I (hs-TpI), serum microRNA quantification (TaqMan PCR analysis, Applied Biosystems, Waltham, MA, USA), and serum MMP-1 and MMP-9 analysis (ELISA assay using specific kits from R&D Systems, Minneapolis, MN, USA). Two-dimensional and three-dimensional echocardiography were used to assess LV ejection fraction; 2D and 3D speckle tracking were used to assess global longitudinal strain (GLS); and echo tracking, CAVI, and peripheral Doppler were used to assess arterial function (Young’s elastic modulus—EP and β index, arterial stiffness, and ABI). The study protocol was approved by the University Hospital of Bucharest Ethics Committee, according to the principles of the Helsinki Declaration with the registration number 392/09.06.2016. All patients signed the written informed consent. 

### 2.2. Sample Collection and RNA Extraction

A venous blood sample was collected from each patient, using an ethylenediaminetetraacetic acid (EDTA) tube of 5 mL. After centrifugation at 1900× *g* for 6 min, the supernatant was transferred to an RNase/DNase-free tube and stored at −80 °C for analysis. Total RNA (included microRNA) was extracted from 400 μL serum with the miRNeasy assay (Qiagen, Hilden, Germany), according to the producer protocol. Before extraction, synthetic cel-miR-39 was added (Applied Biosystems, Thermo Fisher Scientific, Waltham, MA, USA) at a standard concentration, as spike-in control, to evaluate the efficiency of isolation and normalization in the following analysis of RT-qPCR. RNA concentration and its purity were quantified using the spectrophotometer Thermo Scientific™ NanoDrop 2000 (Waltham, MA, USA). RNA was stored at −80 °C until an analysis was made. Complementary DNA was synthetized using the kit High-Capacity cDNA Reverse Transcription Kit—Applied Biosystems (Waltham, MA, USA). The reverse transcription reaction was made using TaqMan™ Gene Expression Master Mix—Applied Biosystems kit and primers TaqMan for the studied microRNAs. The relative level of each microRNA was normalized to cel-miR-39, and calculated using the 2^−Δct^ (ΔCt = Cthsa-miRNA − Ctcel-miR-39) method.

### 2.3. Echocardiography

Each patient was evaluated using a Vivid9 machine (GE Healthcare, Horten, Norway), with M5S and 4 V probes for 2D and 3D echocardiography, respectively. Acquisitions were performed in the left lateral decubitus position, during breath holding, by the same investigator, after blood pressure and heart rate were measured; a one-lead electrocardiogram was used during examination. Three cardiac cycles were achieved at each recording, according to the current recommendations [10]. Images were analyzed offline, using a dedicated software (EchoPac version BT 12 for PC; GE Medical System, Chicago, IL, USA). Left ventricular function was assessed by 2D echocardiography, using the standard parameters: mitral annulus post systolic excursion (MAPSE), Simpson’s biplane LVEF, tissue Doppler analysis for S wave velocity of the lateral and medial mitral annulus, and speckle tracking analysis, at a frame rate of 50–70 fps, in order to obtain apical views (4C, 2C, and 3C) and short-axis views (mitral valve, papillary muscle, and apex). Radial, circumferential, and global longitudinal strain and strain rates were measured. Three-dimensional echocardiography was performed from the apical window, with six consecutive ECG gated volumes and in a 12-slice display mode, with good temporal resolution of all segments, and no stitching artifacts. Data sets stored were analyzed offline using a quantitative software, 4DAutoLVQ (EchoPac version BT 12 for PC; GE Medical Systems). Three-dimensional LVEF and three-dimensional deformation parameters (longitudinal strain, circumferential strain, radial strain, area strain, and global longitudinal strain) were measured. 

### 2.4. Arterial Function

Each patient was evaluated by the echo-tracking system (Aloka Prosound α10, Tokyo, Japan), at the level of the right common carotid artery (RCCA), for which diameter waveform changes were recorded and calibrated for the blood pressure, as an average of five consecutive beats. The following parameters were measured: intima-media thickness (IMT), measured according to the current standards; PWV-E (“single point” pulse wave velocity), calculated from the time delay between two adjacent distension waveforms, using the water hammer equation: PWV-E = (dP/dU)/*ρ* where dP is the difference between systolic and diastolic blood pressure, dU is the difference between arterial diastolic and systolic diameter, and *ρ* is the blood density (1050 kg/m^3^); beta index of stiffness (*β*), calculated according to the formula: *β* = ln (*P*s/*P*d)/[(*D*s − *D*d)/*D*d], where ln is the natural logarithm, *Ps* systolic blood pressure, *P*d diastolic blood pressure, *D*s arterial systolic diameter, and *D*d arterial diastolic diameter; Young modulus of stiffness (*E*p) calculated as: *E*p = (*P*s − *P*d)/[(*D*s − *D*d)/*D*d]; arterial compliance (AC) calculated from the arterial cross area and blood pressure, based on the formula: AC = *π*(*D*s × *D*s − *D*d × *D*d)/[4 × (*P*s − *P*d)]; and augmentation index (AI-E, in %), calculated as AI-E = ΔP/PP [13,14,15,16].

Arterial function was also evaluated by cardio-ankle vascular index (CAVI), PWV, and ankle-brachial index (ABI), obtained in all patients after 5 min of rest in the supine position, using a VaSera CAVI machine (Fukuda Denshi, Tokyo, Japan), with mechanocardiography, electrocardiography, and phonocardiography functions. CAVI is a novel index that assesses stiffness of the aorta, femoral artery, and tibial artery, independently of blood pressure [16]. Cuffs were placed on the upper arms and ankles, electrocardiography electrodes were placed on both wrists, and a phonocardiograph was placed in the aortic space to detect heart sounds. CAVI was automatically determined using the equation: CAVI = a [2*ρ*/(Ps − Pd) × ln(Ps/Pd) × haPWV2] + b where Ps and Pd are systolic and diastolic blood pressure; ρ is blood density; a and b are constants; and PWV is the pulse wave velocity [17]. The heart–ankle PWV was calculated by dividing the distance from aortic valve to ankle, by the sum of time intervals between aortic valve closure sound (first part of the second heart sound) and the notch of the brachial pulse wave, and between the rise of the brachial pulse wave and the ankle pulse wave, with a cut-off value of 13 m/s [18,19,20]. The ABI was calculated by the ratio of the ankle SBP divided by the arm SBP, with a cut-off value of 0.9 and 9 for CAVI [21,22].

### 2.5. Statistical Analysis

Statistical data analysis was performed using SPSS version 19.0 (SPSS, Inc, Chicago, IL, USA). The normal distribution of parameters was evaluated using the Kolmogorov–Smirnov test. Continuous variables were presented as mean ± standard deviation and comparisons of continuous variables were performed using an independent sample *t*-test, while a comparison between categorical variables was performed using Pearson chi-square test. Correlations between two parameters were performed using univariate Pearson correlation and ANCOVA covariance analysis. All statistical tests were two-tailed, and *p* < 0.05 was considered statistically significant.

## 3. Results

### 3.1. Baseline Characteristics

The baseline characteristics of the subjects are presented in Table 1. Heart rate was higher in patients with STEMI. Cardiovascular risk factors were distributed differently, with current smoking and type 2 diabetes being more frequent in patients with STEMI, whereas hypertension and dyslipidemia were more frequent in patients with SIHD (all *p* < 0.05).

### 3.2. Echocardiographic and Arterial Stiffness Parameters

The 2D and 3D echocardiographic parameters of the groups are presented in Table 2. The 2D and 3D LVEF was significantly lower in patients with STEMI, as well as the 2D radial strain (RS) and 2D longitudinal strain (LS). No significant differences were found for the deformation parameters evaluated by 3D echocardiography between the two groups. The arterial stiffness parameters of the two groups (Table 2) were not different either.

### 3.3. Circulating MicroRNAs and MMPs

**Figure 1:**
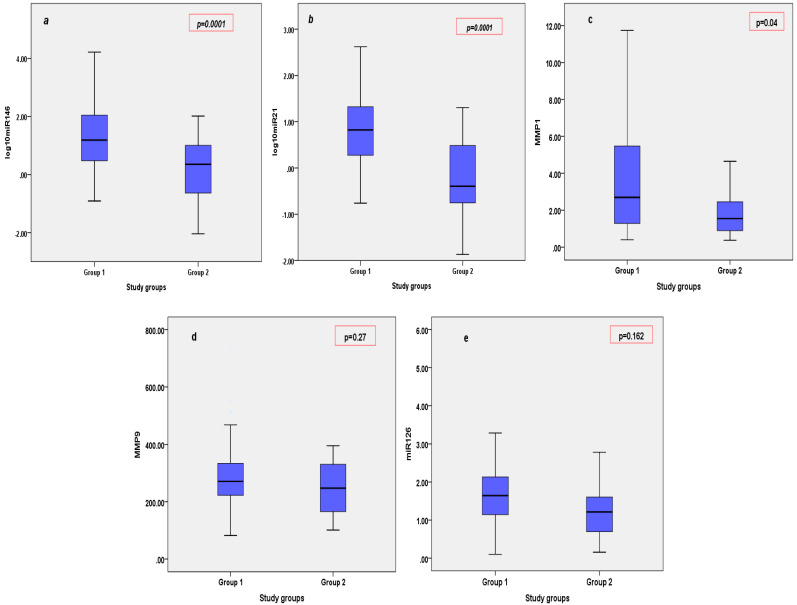
Circulating levels of miRNAs (miRNA146—1**a**, miRNA21—1**b**, miRNA126—1**e**) and MMPs (MMP1—1**c**, MMP9—1**d**) in patients with STEMI (Group 1) vs. SIHD (Group 2).

Circulating levels of miRNA146, miRNA21, and MMP1 were significantly increased in patients with STEMI, by comparison with patients with SIHD (*p* = 0.0001, *p* = 0.0001, *p* = 0.04, respectively) (Figure 1a–c), whereas circulating values of miRNA126 and MMP9 did not differ significantly between patients with STEMI and patients with SIHD (*p* = 0.27, *p* = 0.16, respectively). (Figure 1d,e).

### 3.4. Correlations

**Figure 2:**
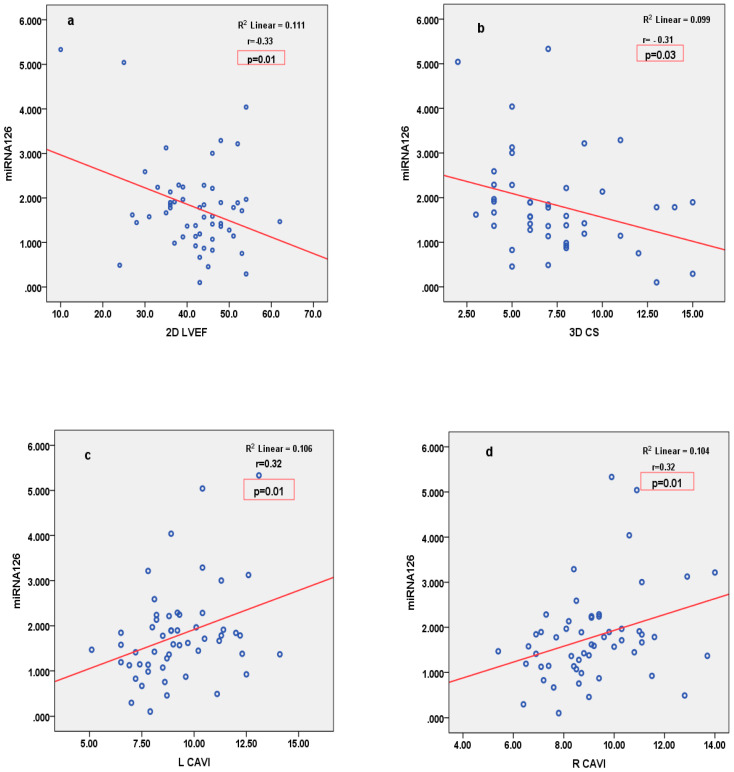
Pearson’s correlations between miRNA126 and 2D LVEF (**a**), 3D CS (**b**), LCAVI (**c**), and RCAVI (**d**) in patients with STEMI. LVEF = left ventricular ejection fraction; CS = circumferential strain; R/L = CAVI right/left cardio-ankle vascular index.

**Figure 3:**
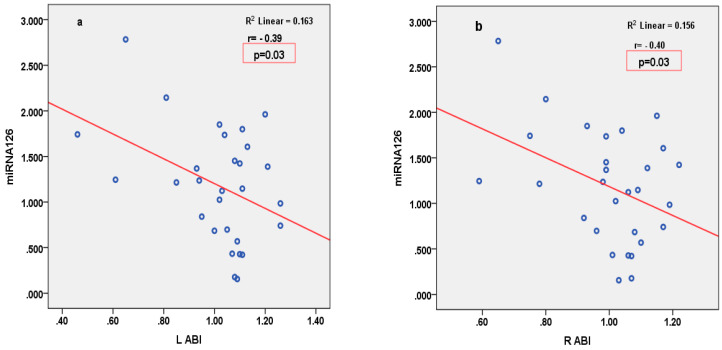
Pearson’s correlations between miRNA126 and LABI (**a**) and RABI (**b**) in patients with SIHD. LABI = left ankle-brachial index; RABI = right ankle-brachial index.

**Figure 4:**
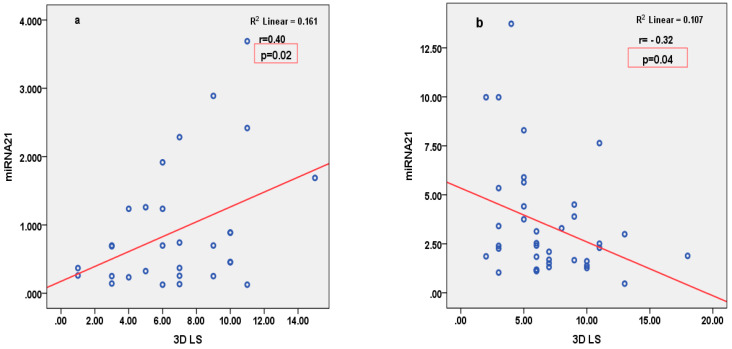
Pearson’s correlations between miRNA21 and 3D LS in patients with SIHD (**a**), and 3D LS in patients with STEMI (**b**). LS = longitudinal strain.

**Figure 5:**
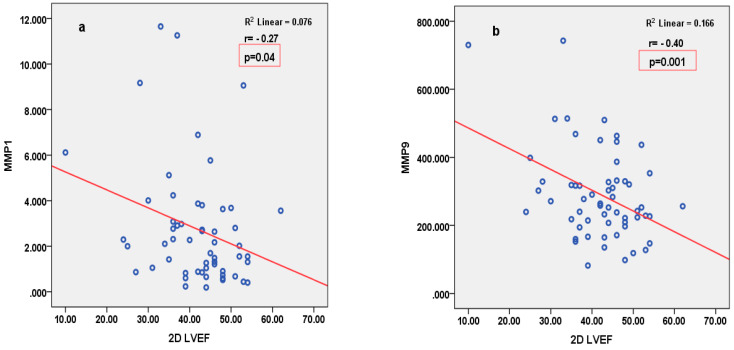
Pearson’s negative correlation between MMP1 (**a**) and MMP9 (**b**) and 2D LVEF in patients with STEMI. LVEF = left ventricular ejection fraction.

**Figure 6:**
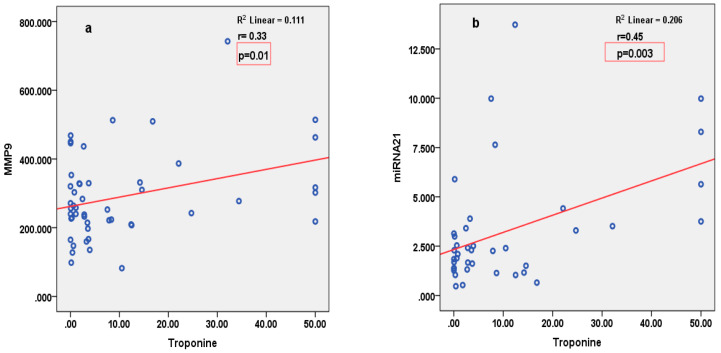
Positive significant correlation between MMP9 (**a**) and miRNA21 (**b**) and troponin in the first 24 h of admission, in patients with STEMI.

miRNA126 correlated inversely with parameters of LV function (2D LVEF and 3D CS), and positively with parameters of arterial stiffness (LCAVI and RCAVI) in patients with STEMI (Figure 2). Conversely, in patients with SIHD, miRNA126 correlated negatively with parameters of arterial stiffness (LABI and RABI) (Figure 3). miRNA21 correlated positively with parameters of LV deformation (3D LS) in patients with SIHD and correlated negatively with 3D LS in patients with STEMI (Figure 4). miRNA146 did not have any significant correlations, with parameters of either LV function or arterial stiffness. Both MMP1 and MMP9 correlated negatively with 2D LVEF and 2D LS in patients with STEMI (Figure 5), but they did not correlate with left ventricle function parameters in patients with SIHD. Meanwhile, MMP9 correlated positively with parameters of arterial stiffness (LCAVI and RCAVI) in patients with SIHD (r = 0.40 and r = 0.32, respectively; both *p* < 0.05). We also analyzed the relation with troponin patients with STEMI; miRNA21 and MMP9 correlated positively with troponin (Figure 6). Using the analysis of covariance (ANCOVA), we performed a linear model for each miRNA and MMP studied using the covariates: smoking, diabetes, hypertension, and dyslipidemia, and it showed no differences in the values of miRNAs and MMPs in patients with STEMI and those with SIHD, compared to an independent *t*-test. We therefore obtained significant higher values of miRNAs and MMPs in STEMI patients compared to SIHD patients independently of the difference in the prevalence of the risk factors.

To provide a comprehensive overview of all correlation analyses, including both positive and non-significant results, we have summarized our findings in Table 3.

## 4. Discussion

In this study, we demonstrated that expression levels of circulating miRNA146 and miRNA21 are up-regulated in patients with STEMI in comparison with patients with SIHD. We also found that higher values of miRNA126 and miRNA21 are associated with lower values of LVEF and lower values of LV strain parameters in patients with STEMI, compared to patients with chronic atherosclerotic disease.

In the literature, more and more studies suggest the role of miRNAs in cardiovascular disease, and their involvement in myocardial infarction and atherosclerosis [23]. Despite this, there is not enough knowledge about the late stages of atherosclerosis, and the process involved in the balance of stable and unstable plaque [24,25]. There are studies that show that circulating miRNAs are specifically expressed in tissues and have a significant role in the pathological process of myocardial infarction [26]. After myocardial infarction, an innate immune mechanism is developed by necrotic cardiomyocytes, through an intense inflammatory reaction. This mechanism is based on Toll-like receptor-mediated pathways, an increase in reactive oxygen production, the up-regulation of cytokines and chemokines, processes that are found to be regulated by circulating miRNAs [27]. In our study, the levels of circulating miRNA126 and miRNA146 were significantly higher in patients with STEMI than in those with SIHD. Increased concentrations of miRNA126 are associated with excessive inflammation, as it has been shown that miRNA126 suppresses inflammatory and necrotic process [28].

Regarding the vascular function, miRNA126 is specifically expressed in vascular endothelial cells and vascular smooth cells, and it is involved in differentiation, apoptosis, and cell proliferation, contributing to the evolution of atherosclerotic plaque [28]. Wang et al. showed that miRNA126 regulates angiogenesis through multiple signaling pathways, and it contributes to neo-angiogenesis and atherosclerosis in coronary artery disease [29]. This could explain our results in patients with SIHD showing that higher values of miRNA126 are negatively correlated with arterial stiffness parameter ABI. Weber et al. showed that the general role of miRNAs in the process of atherosclerosis and inflammation is related to smooth muscle cell function, neointima formation, and lipid metabolism, processes that are more intense in patients with myocardial infarction than in those with chronic coronary disease [30]. Davis et al. showed that miRNA21 can activate and induce a contractile phenotype in human vascular smooth muscle cells, by affecting bone morphogenic proteins in patients with a chronic atherosclerotic process [31]. It has also been shown that lower levels of miRNA21 can facilitate inflammation in cardiac tissue and are responsible for the increased production of inflammatory cytokines in macrophages triggered by damage-associated molecular patterns after myocardial infarction, contributing to the development of cardiac dysfunction and reverse remodeling [32]. Taking into consideration its protective role in cardiomyocytes’ apoptosis and its inhibitory function in inflammation after myocardial necrosis, miRNA 21 might be a novel therapeutic target.

Thum et al. showed that miRNA21 promoted cardiac fibrosis in an experimental heart failure model. They found that miRNA21 levels were high in fibroblasts of the failing heart, increasing the mitogen-activated protein kinase (MAPK) and, therefore, increasing the secretion of growth factors and survival of fibroblasts, leading to extended fibrosis and cardiac hypertrophy [33]. Conversely, injection of a specific miRNA21 antagomir in a mouse model induced a reduction in gene encoding collagens and extracellular matrix, with a reduction in MAPK activity, the inhibition of interstitial fibrosis, and an improvement in cardiac function [33,34]. Endothelial cells have an essential role in the atherosclerosis process and fibrosis, as they regulate the synthesis and secretion of vasoactive substances, platelet aggregation, leukocytes adhesion, and thrombosis. In particular, miRNA21 upregulation has an important role in carotid atherosclerosis, through endothelial cells’ senescence, proliferation, and migration [35]. Moreover, Barwari et al. showed that local delivery of miRNA21 mimics to carotid plaques using ultrasound-targeted microbubble destruction increased plaque stability. They suggest that by increasing smooth muscle cells’ proliferation, miRNA21 mimics might have a stabilizing effect on the fibrous cap that shields the lipid-filled core of atherosclerotic plaques [36]. This might explain the negative correlation, with higher values of miRNA21 in patients with lower left ventricular deformation in patients with STEMI, whereas in patients with SIHD there is a positive correlation.

In our study, both MMP1 and MMP9 had negative correlations with parameters of left ventricular function in patients with STEMI. It has been shown previously that MMP9 is increased in the atherosclerotic active plaque, and it contributes to inflammation and instability of the plaque [37]. The inflammatory cells, like activated macrophages and T cells, together with the active MMPs that lead to the degradation of collagen and elastin, determine the acceleration of the process [38,39]. Meanwhile, in patients with SIHD, we found that MMP9 correlated positively with CAVI values, proving the existence of an active atherosclerotic process and high arterial stiffness in these patients. Furthermore, miRNA21 and MMP9 exhibited a positive correlation with hs-TnI in patients with STEMI, suggesting that they might be novel diagnostic and prognostic biomarkers in STEMI.

Our study takes into consideration the significance of increased miRNA21, miRNA126, and miRNA146, as well as MMP1 and MMP9, that may be considered as potential candidate biomarkers for the early diagnosis of acute myocardial infarction and also for the left ventricular systolic dysfunction as well as plaque vulnerability.

### 4.1. Limitations

The number of patients was relatively small. Analysis was performed only for the baseline parameters, with further follow-up being needed to establish the diagnostic and prognostic potential of these biomarkers. In this study, we did not include a control group, taking into consideration that at this moment, we did not expect any correlation in normal subjects. Further larger studies are planned, which will include a control group. Our aim was to establish the relation of these biomarkers with left ventricle and arterial function, which is hypothesis raising, and further research must be performed in order to establish their prognostic role.

### 4.2. Conclusions

miRNA126, miRNA21, and both MMP1 and MMP9 are potential biomarkers of LV function in STEMI patients, which negatively correlate with LVEF and LV deformation parameters and positively correlate with arterial function parameters. Furthermore, miRNA126 and both MMP1 and MMP9 have a significant positive association with arterial function parameters in patients with SIHD. Circulating miRNAs are promising biomarkers for the early diagnosis and prognosis of acute myocardial infarction. However, further studies are needed to establish their role in cardiovascular diseases.

## Figures and Tables

**Table 1:** Baseline characteristics of the study groups. STEMI = ST-segment elevation myocardial infarction; SIHD = stable ischemic heart disease; BMI = body mass index; SBP = systolic blood pressure; DBP = diastolic blood pressure. Values are shown as mean ± SD or percentage; *p*-value is between STEMI and SIHD.

General Characteristics	STEMI (*n* = 60)	SIHD (*n* = 30)	*p*-Value
Age (years)	57.3 ± 13.4	61.1 ± 10.6	0.19
Male/female (*n*/*n*)	43/17	18/12	0.26
BMI (kg/m^2^)	28.0 ± 4.1	28.5 ± 3.5	0.54
SBP (mmHg)	129.8 ± 22.9	134.3 ± 16.9	0.35
DBP (mmHg)	76.6 ± 9.4	80.2 ± 9.8	0.09
Heart rate (beats/min)	75.9 ± 12.1	67.4 ± 11.5	0.02
Cardiovascular risk factors			
Current smoker (%)	50.0	10.0	0.001
Hypertension (%)	73.3	96.7	0.008
Type 2 diabetes (%)	50.0	26.7	0.035
Dyslipidaemia (%)	88.3	100	0.05

**Table 2:** The 2D and 3D echocardiographic parameters, and arterial stiffness parameters of the study groups. STEMI = ST-segment elevation myocardial infarction; SIHD = stable ischemic heart disease; LVEDV = left ventricular end-diastolic volume; LVESV = left ventricular end-systolic volume; LVEF = left ventricular ejection fraction; MAPSE = mitral annulus post systolic excursion; Smi_m = medial mitral annular S velocity; Smi_l = lateral mitral annular S velocity; RS = radial strain; CS = circumferential strain; LS = longitudinal strain; IMT = intima-media thickness; Ep = Young modulus of stiffness; AC = arterial compliance; AIX = augmentation index; R/L CAVI = right/left cardio-ankle vascular index; R/L ABI = right/left ankle-brachial index. Values are shown as mean ± SD or percentage; *p*-value is between STEMI and SIHD.

Parameter	STEMI (*n* = 60)	SIHD (*n* = 30)	*p*-Value
2D LVEDV (mL)	124.4 ± 34.5	120.4 ± 34.1	0.60
2D LVESV (mL)	71.9 ± 27.6	61.5 ± 23.8	0.08
2D LVEF (%)	42.0 ± 8.9	49.6 ± 7.6	0.0001
MAPSE (mm)	12.5 ± 6	13.6 ± 2.1	0.32
TDI Smi_m (cm/s)	6.8 ± 1.5	6.7 ± 1.2	0.88
TDI Smi_l (cm/s)	7.4 ± 2.3	7.9 ± 2.0	0.28
2D RS (%)	16.1 ± 8.4	21.3 ± 9.0	0.011
2D CS (%)	12.7 ± 3.7	14.3 ± 3.8	0.06
2D LS (%)	10.0 ± 3.8	14.1 ± 3.1	0.0001
3D LVEDV (mL)	111.3 ± 27.7	103.7 ± 28.6	0.25
3D LVESV (mL)	62.9 ± 21.5	52.2 ± 20.0	0.31
3D LVEF (%)	44.3 ± 7.4	50.6 ± 7.2	0.0001
3D RS (%)	16.6 ± 8.9	18.4 ± 8.7	0.37
3D CS (%)	7.4 ± 3.3	8.4 ± 3.6	0.21
3D LS (%)	6.5 ± 3.4	6.8 ± 3.3	0.72
IMT (mm)	0.8 ± 0.2	0.8 ± 0.2	0.52
Ep (kPa)	131.7 ± 56.4	142.7 ± 61.2	0.42
β index	10.0 ± 3.9	11.3 ± 4.8	0.20
AC (mm^2^/kPa)	0.7 ± 0.3	0.7 ± 0.4	0.99
AIX (%)	4.4 ± 24.3	12.3 ± 14.3	0.12
RCAVI	9.3 ± 1.9	8.9 ± 1.7	0.36
LCAVI	9.3 ± 1.9	8.9 ± 1.5	0.32
RABI	0.9 ± 0.17	0.9 ± 0.1	0.92
LABI	1.0 ± 0.1	1.0 ± 0.2	0.98

**Table 3:** Results of the study investigating the correlations between the two study groups. STEMI = ST-segment elevation myocardial infarction; SIHD = stable ischemic heart disease; LVEDV = left ventricular end-diastolic volume; LVESV = left ventricular end-systolic volume; LVEF = left ventricular ejection fraction; MAPSE =mitral annulus post systolic excursion; Smi_m = medial mitral annular S velocity; Smi_l = lateral mitral annular S velocity; RS = radial strain; CS = circumferential strain; LS = longitudinal strain; IMT = intima-media thickness; Ep = Young modulus of stiffness; AC = arterial compliance; AIX = augmentation index; R/L CAVI = right/left cardio-ankle vascular index; R/L ABI = right/left ankle-brachial index. *p*-value is between miR and each parameter studied.

	Parameter	miR126	miR146	miR21	MMP1	MMP9
Pearson correlation (R) and *p*-value	STEMI					
	2D LVEDV (mL)	R = −0.03, *p* = 0.81	R = 0.05, *p* = 0.71	R = 0.13, *p* = 0.36	R = 0.03, *p* = 0.82	R = 0.08, *p* = 0.53
	2D LVESV (mL)	R = 0.01, *p* = 0.94	R = −0.02, *p* = 0.86	R = 0.09, *p* = 0.51	R = 0.11, *p* = 0.38	R = 0.11, *p* = 0.40
	2D LVEF (%)	R = −0.33, *p* = 0.01	R = 0.15, *p* = 0.26	R = −0.07, *p* = 0.63	R = −0.27, *p* = 0.04	R = −0.40, *p* = 0.001
	MAPSE (mm)	R = 0.35, *p* = 0.009	R = −0.02, *p* = 0.86	R = 0.10, *p* = 0.49	R = 0.09, *p* = 0.46	R = 0.33, *p* = 0.009
	TDI Smi_m (cm/s)	R = −0.23, *p* = 0.08	R = 0.17, *p* = 0.23	R = −0.02, *p* = 0.86	R = −0.23, *p* = 0.08	R = −0.14, *p* = 0.29
	TDI Smi_l (cm/s)	R = 0.04, *p* = 0.74	R = 0.25, *p* = 0.06	R = 0.26, *p* = 0.08	R = −0.18, *p* = 0.16	R = −0.03, *p* = 0.79
	2D RS (%)	R = −0.20, *p* = 0.13	R = 0.07, *p* = 0.59	R = 0.02, *p* = 0.89	R = −0.18, *p* = 0.18	R = −0.26, *p* = 0.05
	2D CS (%)	R = −0.04, *p* = 0.73	R = 0.03, *p* = 0.79	R = 0.04, *p* = 0.76	R = −0.02, *p* = 0.83	R = −0.09, *p* = 0.47
	2D LS (%)	R = −0.13, *p* = 0.33	R = 0.02, *p* = 0.85	R = −0.08, *p* = 0.56	R = −0.27, *p* = 0.03	R = −0.26, *p* = 0.04
	3D LVEDV (mL)	R = −0.02, *p* = 0.83	R = 0.00, *p* = 0.99	R = 0.02, *p* = 0.86	R = 0.04, *p* = 0.79	R = 0.08, *p* = 0.58
	3D LVESV (mL)	R = 0.03, *p* = 0.80	R = 0.00, *p* = 0.97	R = 0.07, *p* = 0.63	R = 0.05, *p* = 0.71	R = 0.09, *p* = 0.52
	3D LVEF (%)	R = −0.11, *p* = 0.43	R = −0.02, *p* = 0.86	R = −0.18, *p* = 0.26	R = −0.07, *p* = 0.60	R = −0.14, *p* = 0.33
	3D RS (%)	R = −0.25, *p* = 0.08	R = −0.10, *p* = 0.49	R = −0.28, *p* = 0.08	R = −0.21, *p* = 0.16	R = −0.30, *p* = 0.03
	3D CS (%)	R = −0.31, *p* = 0.03	R = −0.04, *p* = 0.77	R = −0.25, *p* = 0.11	R = −0.19, *p* = 0.19	R = −0.31, *p* = 0.03
	3D LS (%)	R = −0.16, *p* = 0.27	R = −0.18, *p* = 0.23	R = −0.32, *p* = 0.04	R = −0.19, *p* = 0.20	R = −0.22, *p* = 0.12
	IMT (mm)	R = 0.21, *p* = 0.13	R = 0.02, *p* = 0.85	R = −0.17, *p* = 0.29	R = 0.22, *p* = 0.12	R = 0.17, *p* = 0.24
	Ep (kPa)	R = 0.05, *p* = 0.72	R = 0.09, *p* = 0.53	R = 0.15, *p* = 0.32	R = 0.10, *p* = 0.47	R = 0.11, *p* = 0.40
	β index	R = 0.03, *p* = 0.78	R = 0.02, *p* = 0.84	R = −0.33, *p* = 0.01	R = 0.06, *p* = 0.65	R = 0.12, *p* = 0.36
	AC (mm^2^/kPa)	R = −0.09, *p* = 0.52	R = −0.07, *p* = 0.63	R = 0.13, *p* = 0.40	R = −0.06, *p* = 0.66	R = 0.00, *p* = 0.94
	AIX (%)	R = 0.07, *p* = 0.71	R = 0.01, *p* = 0.94	R = −0.21, *p* = 0.17	R = 0.05, *p* = 0.72	R = −0.01, *p* = 0.92
	RCAVI	R = 0.32, *p* = 0.01	R = 0.07, *p* = 0.58	R = 0.07, *p* = 0.62	R = 0.16, *p* = 0.22	R = 0.18, *p* = 0.15
	LCAVI	R = 0.32, *p* = 0.01	R = 0.08, *p* = 0.53	R = 0.15, *p* = 0.29	R = 0.11, *p* = 0.40	R = 0.14, *p* = 0.27
	RABI	R = −0.02, *p* = 0.84	R = 0.17, *p* = 0.23	R = 0.14, *p* = 0.32	R = −0.07, *p* = 0.57	R = 0.01, *p* = 0.93
	LABI	R = −0.02, *p* = 0.83	R = 0.13, *p* = 0.38	R = 0.09, *p* = 0.52	R = −0.11, *p* = 0.41	R = −14, *p* = 0.27
Pearson correlation (R) and *p*-value	SIHD					
	2D LVEDV (mL)	R = 0.07, *p* = 0.78	R = 0.06, *p* = 0.75	R = −0.15, *p* = 0.41	R = −10, *p* = 0.60	R = 0.15, *p* = 0.41
	2D LVESV (mL)	R = −0.04, *p* = 0.98	R = −0.01, *p* = 0.93	R = −0.11, *p* = 0.55	R = 0.05, *p* = 0.80	R = 0.19, *p* = 0.32
	2D LVEF (%)	R = 0.06, *p* = 0.73	R = 0.07, *p* = 0.70	R = 0.04, *p* = 0.98	R = −0.29, *p* = 0.15	R = −0.08, *p* = 0.68
	MAPSE (mm)	R = 0.41, *p* = 0.02	R = 0.23, *p* = 0.24	R = −0.37, *p* = 0.04	R = −0.29, *p* = 0.14	R = −0.04, *p* = 0.82
	TDI Smi_m (cm/s)	R = 0.08, *p* = 0.64	R = 0.24, *p* = 0.22	R = 0.06, *p* = 0.73	R = −0.25, *p* = 0.20	R = 0.03, *p* = 0.87
	TDI Smi_l (cm/s)	R = 0.00, *p* = 0.99	R = 0.00, *p* = 0.98	R = 0.−29, *p* = 0.11	R = −0.09, *p* = 0.65	R = 0.08, *p* = 0.68
	2D RS (%)	R = −0.14, *p* = 0.47	R = 0.11, *p* = 0.56	R = −0.01, *p* = 0.94	R = −0.18, *p* = 0.37	R = −0.21, *p* = 0.29
	2D CS (%)	R = −0.004, *p* = 0.98	R = 0.05, *p* = 0.77	R = −0.13, *p* = 0.47	R = −0.26, *p* = 0.20	R = 0.14, *p* = 0.48
	2D LS (%)	R = 0.23, *p* = 0.21	R = 0.27, *p* = 0.16	R = −0.19, *p* = 0.30	R = −0.11, *p* = 0.56	R = −0.02, *p* = 0.88
	3D LVEDV (mL)	R = −0.03, *p* = 0.86	R = −0.07, *p* = 0.73	R = −0.30, *p* = 0.10	R = −0.14, *p* = 0.48	R = 0.17, *p* = 0.37
	3D LVESV (mL)	R = −0.07, *p* = 0.71	R = −0.14, *p* = 0.47	R = −0.26, *p* = 0.16	R = 0.02, *p* = 0.91	R = 0.23, *p* = 0.23
	3D LVEF (%)	R = 0.07, *p* = 0.71	R = 0.18, *p* = 0.35	R = 0.13, *p* = 0.48	R = −0.28, *p* = 0.16	R = −0.25, *p* = 0.19
	3D RS (%)	R = −0.13, *p* = 0.47	R = 0.24, *p* = 0.21	R = 0.50, *p* = 0.005	R = 0.02, *p* = 0.91	R = −0.06, *p* = 0.76
	3D CS (%)	R = −0.16, *p* = 0.40	R = 0.26, *p* = 0.18	R = 0.46, *p* = 0.009	R = 0.02, *p* = 0.91	R = −0.05, *p* = 0.77
	3D LS (%)	R = −0.05, *p* = 0.78	R = −25, *p* = 0.19	R = 0.40, *p* = 0.02	R = −0.003, *p* = 0.99	R = −0.37, *p* = 0.85
	IMT (mm)	R = 0.24, *p* = 0.23	R = 0.15, *p* = 0.48	R = −0.02, *p* = 0.88	R = 0.18, *p* = 0.40	R = −0.08, *p* = 0.70
	Ep (kPa)	R = −0.21, *p* = 0.29	R = −0.13, *p* = 0.52	R = −0.09, *p* = 0.63	R = 0.17, *p* = 0.42	R = −0.02, *p* = 0.89
	β index	R = −0.18, *p* = 0.37	R = −0.04, *p* = 0.85	R = −0.13, *p* = 0.51	R = 0.08, *p* = 0.69	R = −0.08, *p* = 0.70
	AC (mm^2^/kPa)	R = 0.27, *p* = 0.16	R = 0.18, *p* = 0.37	R = 0.09, *p* = 0.63	R = −0.17, *p* = 0.43	R = −0.13, *p* = 0.51
	AIX (%)	R = −0.001, *p* = 0.99	R = −0.07, *p* = 0.73	R = −0.04, *p* = 0.84	R = −0.26, *p* = 0.21	R = −0.16, *p* = 0.42
	RCAVI	R = −0.25, *p* = 0.18	R = −0.34, *p* = 0.07	R = −0.11, *p* = 0.55	R = 0.16, *p* = 0.41	R = 0.32, *p* = 0.04
	LCAVI	R = −0.27, *p* = 0.15	R = −0.28, *p* = 0.15	R = −0.05, *p* = 0.76	R = 0.09, *p* = 0.63	R = 0.40, *p* = 0.03
	RABI	R = −0.40, *p* = 0.03	R = 0.16, *p* = 0.41	R = 0.07, *p* = 0.70	R = 0.08, *p* = 0.66	R = −0.14, *p* = 0.45
	LABI	R = −0.39, *p* = 0.03	R = 0.17, *p* = 0.37	R = 0.16, *p* = 0.38	R = 0.19, *p* = 0.35	R = −0.06, *p* = 0.76

## Data Availability

All the data processed in this article are part of the research for a doctoral thesis, which is archived in the Cardiology Department at the University and Emergency Hospital of Bucharest. The original data are available upon responsible request.
